# Genetic variants in *CYP2A6* and *UGT1A9* genes associated with urinary nicotine metabolites in young Mexican smokers

**DOI:** 10.1038/s41397-020-0147-4

**Published:** 2020-01-21

**Authors:** Gissela Borrego-Soto, Yadira X. Perez-Paramo, Gang Chen, Sandra K. Santuario-Facio, Jesus Santos-Guzman, Rodolfo Posadas-Valay, Fatima M. Alvarado-Monroy, Isaias Balderas-Renteria, Ramses Medina-Gonzalez, Rocio Ortiz-Lopez, Philip Lazarus, Augusto Rojas-Martinez

**Affiliations:** 1 Tecnologico de Monterrey, Escuela de Medicina y Ciencias de la Salud, Monterrey, Mexico; 2 Department of Pharmaceutical Sciences, College of Pharmacy and Pharmaceutical Sciences, Washington State University, Spokane, WA USA; 3 Facultad de Medicina, Centro Universitario de Salud, Universidad Autonoma de Nuevo Leon, Monterrey, Mexico; 4 Facultad de Ciencias Quimicas, Universidad Autonoma de Nuevo Leon, Monterrey, Mexico; 5 Centro de Investigación y Desarrollo en Ciencias de la Salud, Universidad Autonoma de Nuevo Leon, Monterrey, Mexico

**Keywords:** Genotype, Genetic markers

## Abstract

Nicotine is the major pharmacologically active substance in tobacco. Several studies have examined genotypes related to nicotine metabolism, but few studies have been performed in the Mexican population. The objective was to identify associations between gene variants in metabolizing enzymes and the urinary levels of nicotine metabolites among Mexican smokers. The levels of nicotine and its metabolites were determined in the urine of 88 young smokers from Mexico, and 167 variants in 24 genes associated with nicotine metabolism were genotyped by next-generation sequencing (NGS). *Trans-*3′-hydroxy-cotinine (3HC) and 4-hydroxy-4-(3-pyridyl)-butanoic acid were the most abundant metabolites (35 and 17%, respectively). CYP2A6*12 was associated with 3HC (*p* = 0.014). The rs145014075 was associated with creatinine-adjusted levels of nicotine (*p* = 0.035), while the rs12471326 (*UGT1A9*) was associated to cotinine-*N*-glucuronide (*p* = 0.030). *CYP2A6* and *UGT1A9* variants are associated to nicotine metabolism. 4HPBA metabolite was an abundant urinary metabolite in young Mexican smokers.

## Introduction

Smoking is a risk factor for six of the eight leading causes of death in the world, including lung cancer [1]. The World Health Organization (WHO) estimates that without adequate controls, tobacco-related deaths will increase to more than 8 million in the year 2030, particularly in developing countries [2]. In Mexico, about 10 thousand new cases of lung cancer are diagnosed every year and the majority are related to smoking (71%). Lung cancer ranks seventh in incidence among cancer sites and is first in mortality, and about 8000 Mexicans die from this neoplasm each year [3].

**Fig. 1:**
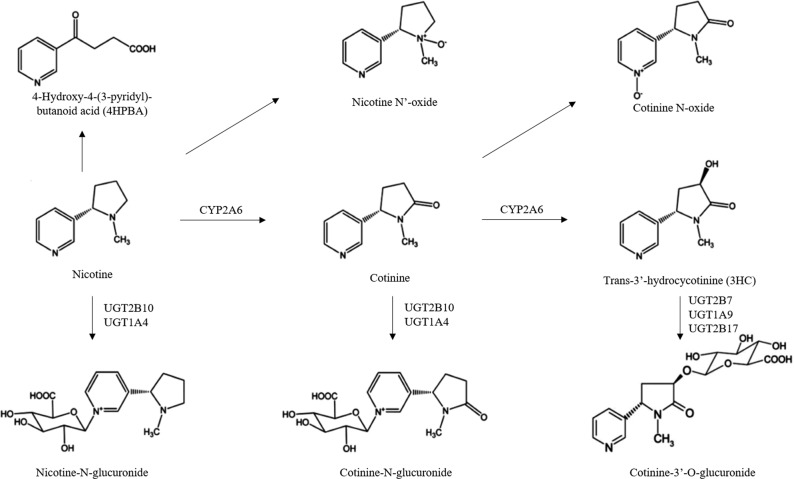
Schematic of major nicotine metabolites (adapted from Perez-Paramo et al. [7]).

Nicotine is associated with tobacco addiction. Most of the nicotine metabolism takes place in the liver [4]. Approximately 80% of nicotine metabolism in the body is by phase I metabolism to cotinine and then to *trans*-3′-hydroxy-cotinine (3HC), mainly by the enzyme CYP2A6. Nicotine, cotinine and 3HC are all glucuronidated in phase II metabolism by the glucuronosyltransferase (UGT) enzymes to render nicotine–*N*-glucuronide (nicotine-Gluc), cotinine-*N*-glucuronide (cotinine-Gluc) and 3HC-*O*-glucuronide (3HC-Gluc; Fig. 1), resulting in more polar molecules that are easily excreted in the urine [5, 6]. Also, nicotine and cotinine are oxidized to nicotine-*N*′-Oxide and cotinine-*N*-Oxide by flavin monooxygenases enzymes (FMOs) [7], and it is believed that 4-oxo-4-(3-pyridyl) butanoic acid (4HPBA) is formed by the hydroxylation of nicotine by the CYP2A6 enzyme [8, 9].

Enzymes of nicotine metabolism are highly polymorphic and their genetic variants can cause differences in enzyme activity [7, 10–16]. This may translate into inter-individual differences in the concentration of nicotine in the body and to differences in the number of cigarettes consumed by smokers [17]. For example, *CYP2A6* reduced activity gene variants are associated with reduced lung cancer risk in smokers [18, 19] and nicotine dependence [20, 21] in different populations. While this variants are associated to increased risk for tobacco-related cancers in humans [22–24], they also can affect smoking-frequency, nicotine clearance, and data interpretation of tobacco use [25]. It has been reported that among Mexican smokers between 18 and 35 years, the average number of cigarettes consumed is 1–5 per day and less than half (37%) smoke daily [26]. This is considerably less than that observed in other populations. For example, about 76% of U.S. smokers consume tobacco every day and 40% of them smoke more than 10–19 cigarettes per day [27]. African American, Caucasians, and Latino subjects older than 25-years-old smoke an average of 9.7 cigarettes per day, and 88% of them report daily smoking for 6 months or more [28].

Single nucleotide polymorphisms (SNPs) have been associated with the metabolism of nicotine in several ethnic groups, but studies in Mexicans are still scarce [21, 29]. The objective of the present study was to determine whether associations exist between genetic variants in nicotine metabolizing enzyme genes and the concentration of urinary metabolites of nicotine in Mexican smokers.

## Material and methods

### Study population

The study protocol was approved by the Ethics Committee of the School of Medicine and the University Hospital of the Universidad Autonoma de Nuevo Leon (code BI12-005) and ethical standards of the Declaration of Helsinki were followed. Questionnaire data and samples were collected from 88 university students from the northeast region of Mexico. This *n* was adapted to the number of cells available for NGS in the TrueSeq Custom amplicon kit (Illumina Inc.). Inclusion criteria included being of ages 18–35 years and having smoked more than five cigarettes in the last 3 days. Informed consent was provided by all subjects. Exclusion criteria included liver or kidney disease and pregnancy. Participants signed an informed consent letter, answered a questionnaire about socio-demographic data and their smoking exposure, and provided 5 mL of peripheral blood anticoagulated with EDTA and 50 mL of urine. Samples were transported to the laboratory at 4 °C within 3 h after sample collection. Genomic DNA was extracted with the TSNT technique (lysis buffer: 2% Triton, 1% SDS, 100 mM NaCl, 10 mM Tris-HCl, pH 8.0), followed by phenol-chloroform extraction. Urine was aliquoted, and samples stored at −20 °C until analysis.

### Quantification of urine nicotine metabolites

Nicotine metabolites were examined using an Acquity liquid chromatography—tandem mass spectrometer (LC-MS/MS system) (Waters Corporation, Milford, MA) as described by Chen et al. [15]. Quantification was performed for the following urinary metabolites: nicotine, nicotine-*N’*-Oxide, nicotine-Gluc, cotinine, cotinine-Gluc, cotinine-*N*-Oxide, 3HC, 3HC-Gluc and 4HPBA. Urinary creatinine was measured as a control for urine secretion and for normalization of nicotine metabolite levels between specimens [15]. To corroborate measurements, repeat testing was performed for 15% of all samples, chosen randomly. The limit of quantitation (LLOQ) of all metabolites are shown in the Table S1. The investigators who performed the phenotyping analysis were blinded to all demographic and genotyping data corresponding to the individual subjects including levels of smoking.

### Quantification of plasma cotinine

Quantification of plasma cotinine was performed in duplicate by ELISA (Calbiotech Inc., CA). The assay’s lower limit of quantitation (LLOQ) was 0.44 ng/mL.

### Genotyping by next-generation sequencing (NGS)

A custom panel was designed to sequence 384 genetic variants in 52 genes related to xenobiotic metabolism (Table S2), which included 167 genetic variants in 24 genes associated with nicotine metabolism. Specific oligonucleotides were designed using Illumina’s Design Studio program (Illumina Inc., San Diego, CA) and the quality and the Illumina staff verified feasibility of their design. A group of 88 subjects who smoked more than five cigarettes in the last 3 days were included. To confirm their tobacco use, plasma cotinine was measured for each subject, with plasma cotinine concentrations ranged between 2.4 and 500 ng/μL. DNA quality and concentrations were evaluated using a Nanodrop 8000 spectrophotometer (260/280 absorbance ratio ≥1.8; Thermo Scientific, Wilmington, DE). NGS libraries were amplified using 250 ng of genomic DNA as template and the Truseq Custom amplicon kit in an Eppendorf EP Master Faster thermocycler (Foster City, CA). The quality of the libraries were evaluated in an Agilent 2100 Bioanalyzer (Santa Clara, CA) and were standardized according to the manufacturer’s instructions. Libraries were adjusted to a concentration of 12 pM and verified using the Quant-iT Picogreen kit (Thermo Scientific). A pool of libraries from 88 subjects was generated and added to the cartridge MiSeq reagent kit V2 (Illumina Inc.) and was sequenced using the platform MiSeq by Illumina (150 bp paired-end reads) (Illumina Inc.). The variant call format files (VCFs) of each sample were visualized in the Variant Studio software (Illumina Inc.).

### CYP2A6*12 genotyping

CYP2A6*12 is a variant resulting from a translocation between the *CYP2A6* and *CYP2A7* genes generated by a *CYP2A6/CYP2A7* hybrid formed by exons 1–2 of the *CYP2A7* gene and exons 3–9 of the *CYP2A6* gene; this causes the loss of 10 amino acids and reduced *CYP2A6* activity [30]. The genotyping of CYP2A6*12 was developed by the method described by Oscarson et al. [30], with small variations. The process was carried out in two steps (PCR1 and PCR2). In PCR1, a common region of CYP2A6*1 (wild type) and CYP1A6*12 (variant) of 2.3 kb was amplified using 100 ng of DNA in each reaction. The reaction mixture was incubated with GoTaq Green Master Mix (Promega) and 2.5 μM of the primers 2A-F (5′-GCACCCCTCCTGAGGTACCAC-3′) and 2A6ex3R1 (5′-GTCCCCTGCTCACCGCCA-3′) in a final volume of 10 μL. The PCR program started with denaturation at 95 °C for 1 min, followed by 35 cycles of denaturation at 95 °C for 15 sec, alignment at 60 °C for 20 s, extension at 72 °C for 1 min and final extension to 72 °C for 7 min. The reaction mixture for PCR2 is a specific allele, the primer 2A6ex1 anneals the exon 1 from *CYP2A6* gene and the primer 2A7ex1 anneals the exón 1 from *CYP2A7* gene. This PCR identifies the wild type allele and the chimeric *CYP2A6/CYP2A7* allele (respectively), both generate an amplicon of approximately 1.5 kb. PCR2 was carried out with 1 μL of a 1:100 dilution of the PCR1 product, GoTaq Green Master Mix (Promega), 50 μM of sense primers (2A6ex1: 5′- AACACAGAGCAGATGTACA-3′ or 2A7ex1: 5′-AACACAGAGCACATATGTG-3′) and 50 μM of antisense primers (5′-CGCTCCCCGTT-GCTGAATA-3′) in a final volume of 10 μL. The PCR program started with a denaturation at 95 °C for 1 min, followed by 30 cycles of denaturation at 95 °C for 15 s, alignment at 60 °C for 20 s, extension at 72 °C for 1 min and a final extension at 72 °C for 7 min. The products were stained with ethidium bromide and visualized in 1.2% agarose gels.

### Data analysis

Total nicotine metabolite levels, or “equivalents” (Total-Nic-Eq) present in each urine sample were determined as the sum of the uM concentrations of nicotine, nicotine-Gluc, nicotine-*N*’-Oxide, cotinine, cotinine-Gluc, cotinine-*N*-Oxide, 3HC, 3HC-Gluc and 4HPBA. The percentage of each analyte in the Total-Nic-Eq and as creatinine-normalized levels was calculated based on the equation: *C* (μM)/[Total-Nic-Eq (uM)] × 100, where “*C*” is the total urinary metabolite concentration. R package (R-project.org) was used to produce the correlation matrix and Pearson analyses to assess relationship among metabolites. Non parametric Spearman correlation was used to compare urinary and plasma cotinine levels with GraphPad Prism software. To describe the study population, we used basic descriptive statistics. For the analysis of the NGS data, The Human Genome Build construct (hg19) was used as a reference [GRCh38.p12. Homo Sapiens (human). Genome Reference Consortium (December 2017). RefSeq assembly: GCF_000001405.38]. The alignment and the variant calling were done with the Miseq Reporter TruSeq Amplicon (Illumina Inc.). The variants were identified and visualized using Variant Studio software (Illumina Inc.), and the data filtering pipeline was as follows: Q > 30, quality of the readings > 100, depth > 60, and allelic frequency > 25%. For the gene and metabolite analysis, the variances between groups were different and a Mann–Whitney test for STATA (StataCorp. 2019. Stata Statistical Software: Release 15. College Station, TX: StataCorp LLC) was used. Significance level for *p*-values was set at 0.05 or less.

## Results

Of the 88 subjects recruited into this study the majority of subjects were native to the state of Nuevo Leon (74%), 24 (27%) were women. The average age of the subjects was 20 years and the median age of smoking initiation was 15 years. For this study, we selected the participants among those subjects who smoked more than five cigarettes in the last 3 days, since in a previous national survey, the range of consumption reported was 1 to 5 cigarettes per day [31]. Seventy-four subjects (84%) indicated that they were daily smokers. Plasma cotinine was proportional to the number of cigarettes smoked (groups of cigarettes smoked: 1–5, 6–10, 11–20, >20; see Table 1). In addition, urinary and plasma cotinine levels show a positive correlation (*r* = 0.79, *p* < 0.0001, and *r*^2^ = 0.50; Fig. S1).

**Table 1:** Demographic characteristics, smoking habits, and plasma cotinine levels in study subjects.

Characteristics	Values	*N* (%)		*P*
*N* total		88		
Origin	Nuevo leon	65 (74)		
	Coahuila	9 (10)		
	Others	14 (16)		
Gender	Female	24 (27)		<0.0001*
	Male	64 (73)		
Smoking daily	Yes	74 (84)		<0.0001*
	No	14 (16)		

Characteristics	Values	*N* (%)	Cotinine (ng/mL) Mean (standard error)	*P*
Number of cigarettes smoked in the last three days	1–5	18 (20)	25 (12.26)	0.007**
	6–10	19 (22)	78 (22)	
	11–15	17 (19)	63 (24)	
	16–20	8 (9)	139 (57)	
	>20	26 (30)	131 (24)	

*P-value from Chi^2^ test

**P-value from ANOVA test

Table 2 shows the levels of each of the urinary nicotine metabolites expressed as a percentage of Total-Nic-Eq and after normalization with creatinine concentration. The major metabolite in smokers was 3HC (35% of Total-Nic-Eq) followed by 4HPBA (17% of Total-Nic-Eq) > 3HC-Gluc (14% of Total-Nic-Eq) > cotinine (9.5% of Total-Nic-Eq) > nicotine-*N*’-Oxide (7.6% of Total-Nic-Eq) > cotinine-Gluc (7.3% of Total-Nic-Eq) > nicotine (4.3% of Total-Nic-Eq) > cotinine-*N*-Oxide (3.4% of Total-Nic-Eq) > nicotine–Gluc (3.0% of Total-Nic-Eq). For the glucuronides, the ratios of 3HC-Gluc were 1.9 and 4.6 times higher than those observed for cotinine-Gluc and nicotine-Gluc, respectively. For the oxidation products, the cotinine-*N*-Oxide ratios were 2.2 times higher than that observed for nicotine-*N*’-Oxide. The levels of 3HC and 4HPBA were 3.7- and 1.7-fold higher, respectively, than those observed for cotinine. When smokers were stratified by nicotine metabolic ratio (NMR), using the previously reported threshold (slow metabolizers < 0.31 and normal and fast metabolizers ≥ 0.31) 98.8% (*n* = 87) of our smokers fell into the normal metabolizers category [32].

**Table 2:** Profile concentration of nicotine metabolites in urine of 88 smokers, expressed in percentage of Total-Nic-Eq and adjusted levels with milligrams of urinary creatinine.

	% de Total-Nic-Eq		Creatinine-adjusted levels (nmol/mg creatinine)	
	Mean ± ES (95% CI)	Range	Mean ± ES (95% CI)	Range
3HC	35 ± 1.6 (32–38)	0.3–61	11 ± 1.2 (8.7–14)	0.02–53
4HPBA	17 ± 2.1 (13–21)	1.6–89	3.9 ± 0.5 (3.0–4.7)	0.02–22
3HC-Gluc	14 ± 0.7 (14–16)	0.1–30	4.4 ± 0.6 (3.2–5.6)	0.004–38
Cotinine	9.5 ± 0.5 (8.5–11)	0.4–24	3.1 ± 0.4 (2.3–3.8)	0.001–26
Nicotine-*N*’-Oxide	7.5 ± 1.4 (4.7–11)	0.7–87	7.2 ± 5.1 (2.7–17)	0.04–447
Cotinine-Gluc	7.0 ± 4.1 (6.4–8.1)	0.3–18	2.5 ± 0.3 (1.9–3.1)	0.01–14
Nicotine	4.0 ± 6.6 (3.0–5.7)	0.1–47	1.2 ± 0.1 (0.9–1.5)	0.001–6.2
Cotinine-*N*-Oxide	3.0 ± 1.6 (3.1–3.7)	0.1–8.2	1.1 ± 0.1 (0.8–1.3)	0.00–6.1
Nicotine-Gluc	3.0 ± 2.5 (2.5–3.5)	0.2–16	1.1 ± 0.2 (0.8–1.5)	0.8–1.4

**Fig. 2:**
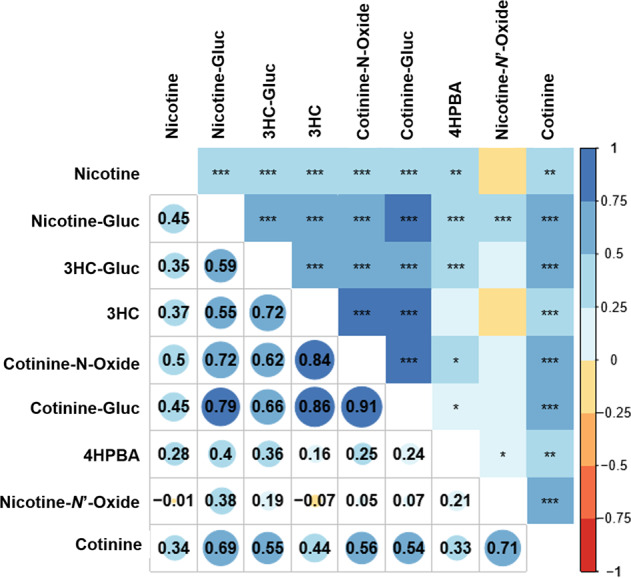
Correlation Matrix of urinary nicotine and its eight major metabolites (nicotine, nicotine-*N*’-oxide, nicotine-Gluc, cotinine, cotinine-*N*-oxide, cotinine-Gluc, 3HC, 3HC-Gluc, and 4HPBA). Each cell in the table shows the correlation between two variables using the color code from the right side, where red indicates negative correlation values and blue indicate positive correlation values. The calculated values of the correlation coefficient are shown in the lower half at below the diagonal of the matrix. Pearson’s product moment correlation coefficient are shown in the upper half above the diagonal of the matrix, where one, two or three asterisks indicate that the *p*-value was lower than 0.05, 0.01, and 0.001, respectively. The empty cells indicate no correlation.

A linear correlation analysis of urinary metabolites normalized by creatinine levels was performed. Figure 2 shows a positive correlation between cotinine-Gluc vs cotinine-*N*-Oxide (*r* = 0.91; *p* < 0.0001), cotinine-Gluc vs 3HC (*r* = 0.86; *p* < 0.0001) and cotinine-*N*-Oxide vs 3HC (*r* = 0.84; *p* < 0.0001). Nicotine-*N*’-Oxide was the least correlated with all metabolites (*r* ≤ 0.38).

A total of 10,283,992 readings were generated per NGS. 95% had a score higher than Q30. 402 SNPs in 68 genes were identified, of which 260 (65%) were found in exons. Of the variants identified, 71 (27%) were missense variants. After filtering sequence data, 11 variants in *CYP2A6*, 14 in *CYP2B6*, 4 in *UGT2B7* and 42 in *UGT1A* isoforms were available for association studies. The variants associated with different concentrations of nicotine metabolites were: CYP2A6*12 and rs145014075 in the *CYP2A6* gene, and rs12471326 in the *UGT1A9* gene locus. These gene variants were in Hardy–Weinberg equilibrium, and the frequencies observed in our studied population are shown in Table 3.

**Table 3:** Allelic and genotypic frequencies of 88 subjects analyzed by NGS.

Variants	Allele 1	Allele 2	Homozygous wild-type	Heterozygotes	Homozygous variant	MAF*	HWE** (*p*)
CYP2A6*12	CYP2A6 0.96	CYP2A6/7 0.04	0.92	0.08	0	0.04	0.67
*CYP2A6* rs145014075	G: 0.97	T: 0.03	0.93	0.07	0	0.03	0.71
*UGT1A9* rs12471326	T: 0.91	C: 0.09	0.82	0.18	0	0.09	0.34

*MAF minor allele frequency.

**HWE Hardy–Weinberg Equilibrium.

**Fig. 3:**
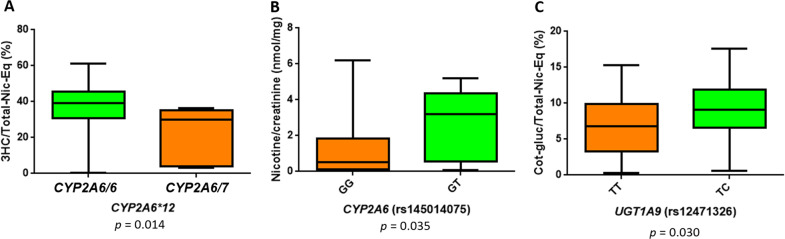
Effect of variants on nicotine metabolite. **a** Genotypes *CYP2A6/6* and *CYP2A6/7* of the *CYP2A6* gene vs percentage of 3HC analyte in the Total-Nic-Eq. **b** GG and GT genotypes of the rs145014075 variant of *CYP2A6* vs creatinine-adjusted levels of nicotine. **c** TT and TC genotypes of the variant rs12471326 of the *UGT1A9* gene vs percentage of cotinine-Gluc analyte in the Total-Nic-Eq. A Mann–Whitney test for STATA (College station, TX) was used to make the comparisons of the genotypes and the levels of the metabolites. Metabolite levels are shown as percentage of analyte in the Total-Nic-Eq and creatinine-adjusted levels and are shown as the mean ± standard error.

The variants CYP2A6*12 and rs145014075, both in the *CYP2A6* gene, were associated with altered levels of 3HC and nicotine, respectively. The variant CYP2A6*12 confers reduced enzyme activity as described above [30] and the variant rs145014075 is a nonsense G/T change generating a stop codon (Ser467Stop). The heterozygous genotype for CYP2A6*12 was associated with low levels of 3HC (*p* = 0.014), 30% of Total-Nic-Eq of cotinine-Gluc were observed in heterozygotes vs 39% in wild type homozygotes. The heterozygous genotype GT of rs145014075 variant was associated with high creatinine-adjusted levels of nicotine (*p* = 0.035): 3% in heterozygotes vs 0.5% in wild type homozygotes (Fig. 3). No homozygotes for the mutant allele were found for either of these variants. The variant rs12471326 in the *UGT1A9* gene was associated with high levels of cotinine-Gluc (*p* = 0.030), 9% of Total-Nic-Eq of cotinine-Gluc was observed in heterozygotes vs 7% in wild type homozygotes (Fig. 3). No homozygotes for the mutant allele were found for this variant.

## Discussion

This study showed that variants in *CYP2A6* are associated to urinary levels of nicotine and 3HC, while the *UGT1A9* variant is associated with urinary levels of cotinine-Gluc in young smokers from northeastern Mexico. The remaining variants in genes related to the nicotine metabolism failed to show associations. Regarding the *CYP2A6* variants, Bloom et al. reported that the rs1137115 and rs28399435 were associated with low metabolism of nicotine in European Americans [33], but in our study no differences were observed among urinary levels of nicotine, cotinine and 3HC with these variants. The variant rs28399433 has been reported to be associated with smoking dependence in Mexican population [34], but no association was observed in our study at large. Nevertheless, when stratifying our samples according to their NMR, only the single sample that fell into the slow metabolizer category (NMR < 0.31) presented with a heterozygous genotype for the rs28399433 variant. The low frequency of this variant among our studied population (MAF = 0.13) might explain the lack of significant associations. CYP2A6 and CYP2B6 are responsible for 90 and 10% of the conversion of nicotine to cotinine, respectively [35]. CYP2A6 is responsible for 100% of the conversion of cotinine to 3HC [6]. Previous studies in cell lines and in vivo have shown that the CYP2A6*12 variant reduces the metabolism of coumarin in approximately 60% of subjects [30, 36]. The CYP2A6*12 variant has been strongly associated with smoking status in Spaniards [37]. In the present study, the heterozygous genotype *CYP2A6/7* was associated with low levels of 3HC as a percentage of the Total-Nic-Eq in young smokers. No differences of Total-Nic-Eq for cotinine were observed in *CYP2A6/6* homozygotes and *CYP2A6/7* heterozygotes. The CYP2A6*12 frequency in our population was low (4%), close to those reported for Spaniard and Chinese populations (2.2 and 0%, respectively) [30]. This variant can predispose to lower consumption of cigarettes [38], but we did not find significant differences in the number of consumed cigarettes in this study (*p* = 0.69).

The variant rs145014075 of the *CYP2A6* gene was found in 3% of the subjects examined in this study. This variant causes a stop codon (Ser467Stop), but functional studies have not yet been reported for the enzymatic effects of the nonsense variant. This variant is reported in the 1000 Genomes Project Database at frequencies of 4% and 2% in African American and European American populations, respectively. In the present study, this variant was associated with high creatinine-adjusted levels of nicotine, although no association was observed when examined as a percentage of Total-Nic-Eq. This association was not observed for the other analyzed variants. While this variant may reduce the activity of CYP2A6 and may be related to lower consumption of cigarettes, no association with the number of consumed cigarettes was found in the present study.

Regarding *UGT2B7* gene variants, an important enzyme for the glucuronidation of 3HC [39], we did not find associations with glucuronides of nicotine metabolites. In the case of *UGT2B17*, most of the variants were discarded after filtering with the parameters described above. We found an association between the levels of urinary cotinine-Gluc and the rs12471326 gene variant of *UGT1A9*. This intronic variant was found to be associated with high percentages of Total-Nic-Eq of cotinine-Gluc. There is a clinical study for this variant showing no differences in the in the postnatal metabolism of bilirubin, but no functional analyses in the nicotine metabolism have been reported [40].

Kaivosaari et al. reported that UGT1A9 did not exhibit any nicotine or cotinine glucuronidation activity in human liver microsomes (HLM) [41], and other studies showed that UGTs 2B10 and 1A4 were the major enzymes responsible for nicotine and cotinine glucuronidation [14]. However, Kuehl and Murphy studies in human liver microsomes suggested that UGT1A9 may also be involved in nicotine and cotinine glucuronidation, particularly contributing to a greater percentage of glucuronidation when nicotine levels are low [42]. The results of this report may be applied to the present study, since the cigarette consumption in the analyzed population is low (1–5 cigarettes per day) when compared with other populations (Latinos: 9.4 cigarettes per day; African Americans:11 cigarettes per day; Native Hawaiians: 15 cigarettes per day) [43].

The ethnicity and age of the studied subjects may explain the lack of associations among nicotine metabolites and variants in other UGTs involved in nicotine metabolism, like UGT2B17, UGT2B7, and UGT1A4, as reported in other studies [39, 44]. Expression of these UGTs may be related to the age, and most of the studies of nicotine metabolism has been performed in elderly subjects [45–47]. The expression of UGT1A9 is age-dependent. Miyagi et al. showed that the activity of UGT1A9 is higher in children than in adults and that this activity may be modulated by post-transcriptional and/or allosteric mechanisms in adults [48].

UGT2B10 is the major enzyme involved in both nicotine-Gluc and cotinine-Gluc formation and variants of this gene affect the glucuronidation of nicotine and cotinine [12, 45, 49]. We did not assess variants for *UGT2B10* and/or for the *FMO* genes and this may affect the interpretation of glucuronidation rates for this study.

Allele frequencies of *UGT1A* variants differ among populations, as observed in the 1000 Genomes Project database. The allele C of rs12471326 variant in *UGT1A9* described here was similar (9%) to that observed for MXL (6%), IBS (2%), CEU (2%), CHB (0%), and YRI (7%).

The levels of nicotine and its eight major metabolites were determined simultaneously in the urine of smokers in the present study. Interestingly, 4HPBA was the second highest metabolite as a percentage of Total-Nic-Eq (17%). Chen et al. analyzed the same metabolites in the Caucasian population using the same methodology and found levels of 4HPBA that were almost 2-fold lower (%Total-Nic-Eq = 8.9%) than the present study [15]. Rangia et al. reported 1.7-fold lower %Total-Nic-Eq of 4HPBA (9.7%) using a one-time urine collection [8], while Piller et al. reported 3.4-fold lower %Total-Nic-Eq (5.0%) in a 24-h urine specimens [50]. Subject age and/or ethnicity as well as level of cigarette consumption could all potentially be relevant factors influencing 4HPBA urinary levels in the population examined in the present study.

In conclusion, genetic variants rs145014075 and CYP2A6*12 in the *CYP2A6* gene were associated with levels of phase I urinary nicotine metabolites and rs12471326 in *UGT1A9* gene were associated with cotinine-Gluc metabolites levels of II phase nicotine metabolites in young Mexican smokers. These variants could be associated with addictive behaviors and with the development of diseases related to smoking in this population. Functional studies are necessary to define the participation of these genetic variants in the nicotine detoxification process. In addition, high levels of 4HPBA were found in the urine of the studied smokers. It has been proposed that CYP2A6 participates in the 2′-hydroxylation of nicotine to produce 4HBPA [9], but this has not been fully evaluated. In the present study, several functional variants of CYP2A6 were analyzed, but none of these correlated with different levels of 4HPBA expressed in both, Total-Nic-Eq or creatinine-adjusted levels. These data are consistent with possibility that other enzymes in addition to CYP2A6 are involved in 4HPBA formation.

Studies characterizing the enzymes involved in 4HPBA formation should be performed to better define the possible health risks related to this metabolite.

## Supplementary information

Figure S1

Table S1

Table S2
