# Supplementary figures and images for: Genetic variants in CYP2A6 and UGT1A9 genes associated with urinary nicotine metabolites in young Mexican smokers
Source: Pharmacogenomics J. 2020 Jan 21;20(4):586–94. doi: 10.1038/s41397-020-0147-4 (PMC7375952; doi:10.1038/s41397-020-0147-4)

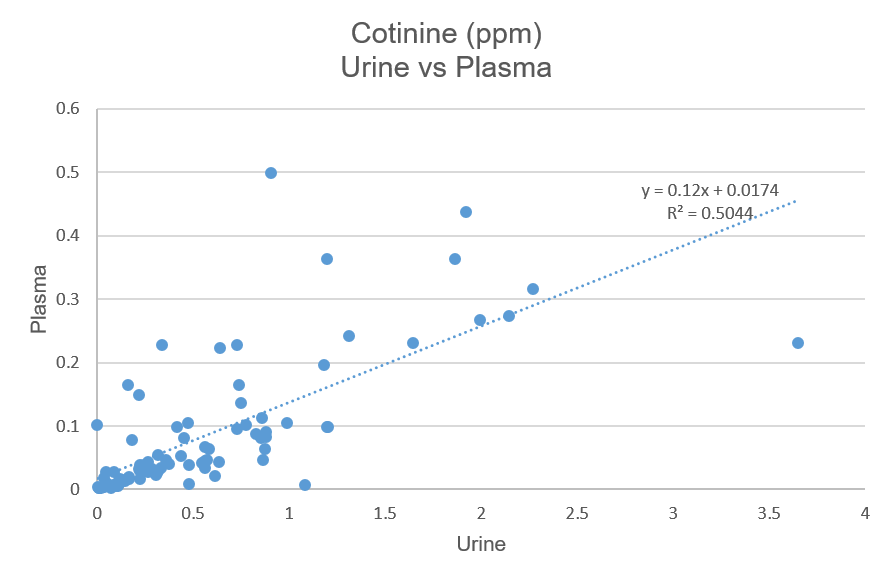

Supplement: Supplementary file 1 — Figure S1 [file 41397_2020_147_MOESM1_ESM.tif]
