# Supplementary material for: Genetic variants in CYP2A6 and UGT1A9 genes associated with urinary nicotine metabolites in young Mexican smokers
Source: Pharmacogenomics J. 2020 Jan 21;20(4):586–94. doi: 10.1038/s41397-020-0147-4 (PMC7375952; doi:10.1038/s41397-020-0147-4)
Supplement: Supplementary file 2 — Table S1 [file 41397_2020_147_MOESM2_ESM.docx]

|  | **Nicotine** | **Nicotine-*N*'-Oxide** | **Nicotine-Gluc** | **Cotinine** | **Cotinine-N-Oxide** | **Cotinine-Gluc** | **3HC** | **3HC-Gluc** | **HPBA** |
| --- | --- | --- | --- | --- | --- | --- | --- | --- | --- |
| ppm | 0.00011 | 0.0011 | 0.0011 | 0.00019 | 0.00006 | 0.00095 | 0.0079 | 0.0027 | 0.0076 |
| µM | 0.00068 | 0.0064 | 0.0032 | 0.0011 | 0.00031 | 0.0027 | 0.041 | 0.0072 | 0.042 |

**Table S1.** Limit of quantitation (LLOQ) of nicotine metabolites.
