# Supplementary material for: Genetic variants in CYP2A6 and UGT1A9 genes associated with urinary nicotine metabolites in young Mexican smokers
Source: Pharmacogenomics J. 2020 Jan 21;20(4):586–94. doi: 10.1038/s41397-020-0147-4 (PMC7375952; doi:10.1038/s41397-020-0147-4)
Supplement: Supplementary file 3 — Table S2 [file 41397_2020_147_MOESM3_ESM.docx]

| **Gene**  **Table S2.** Genetic variants analyzed by NGS. | **Variant** | **Chromosome** | **Localization** |
| --- | --- | --- | --- |
| CYP2A6 | rs28399468 | 19 | 41349732 |
| CYP2A6 | rs5031016 | 19 | 41349774 |
| CYP2A6 | rs8192729 | 19 | 41350996 |
| CYP2A6 | rs72547582 | 19 | 41351200 |
| CYP2A6 | rs28399454 | 19 | 41351267 |
| CYP2A6 | rs2644905 | 19 | 41351967 |
| CYP2A6 | rs4079369 | 19 | 41352762 |
| CYP2A6 | rs3891219 | 19 | 41352912 |
| CYP2A6 | rs28399444 | 19 | 41354190 |
| CYP2A6 | rs28399442 | 19 | 41354458 |
| CYP2A6 | rs1801272 | 19 | 41354533 |
| CYP2A6 | rs4986891 | 19 | 41354629 |
| CYP2A6 | rs1137115 | 19 | 41356281 |
| CYP2A6 | rs8192720 | 19 | 41356310 |
| CYP2A6 | rs28399434 | 19 | 41356319 |
| CYP2A6 | rs28399433 | 19 | 41356379 |
| CYP2A6 | rs28399447 | 19 | 41384826 |
| CYP2A6 | rs4803381 | 19 | 41389129 |
| CYP2B6 | rs34223104 | 19 | 41497129 |
| CYP2B6 | rs8192709 | 19 | 41497274 |
| CYP2B6 | rs35303484 | 19 | 41497346 |
| CYP2B6 | rs36060847 | 19 | 41510030 |
| CYP2B6 | rs12721655 | 19 | 41510282 |
| CYP2B6 | rs35773040 | 19 | 41510286 |
| CYP2B6 | rs4803418 | 19 | 41511803 |
| CYP2B6 | rs4803419 | 19 | 41512792 |
| CYP2B6 | rs3826711 | 19 | 41512824 |
| CYP2B6 | rs36056539 | 19 | 41512828 |
| CYP2B6 | rs3745274 | 19 | 41512841 |
| CYP2B6 | rs36079186 | 19 | 41512918 |
| CYP2B6 | rs45482602 | 19 | 41515255 |
| CYP2B6 | rs2279344 | 19 | 41515483 |
| CYP2B6 | rs28399499 | 19 | 41518221 |
| CYP2B6 | rs34826503 | 19 | 41518244 |
| CYP2B6 | rs34097093 | 19 | 41518370 |
| CYP2B6 | rs35979566 | 19 | 41518598 |
| CYP2B6 | rs35010098 | 19 | 41518708 |
| CYP2B6 | rs8192719 | 19 | 41518773 |
| CYP2A13 | rs1645691 | 19 | 41596742 |
| CYP2A13 | rs3885816 | 19 | 41599665 |
| CYP2A13 | rs1645694 | 19 | 41600808 |
| CYP2A13 | rs1709082 | 19 | 41601609 |
| CYP7A1 | rs8192879 | 8 | 59403576 |
| CYP7A1 | rs8192875 | 8 | 59407065 |
| CYP7A1 | rs8192874 | 8 | 59409373 |
| CYP7A1 | rs3808607 | 8 | 59412924 |
| CYP7A1 | rs12542233 | 8 | 59414401 |
| CYP7A1 | rs881671 | 8 | 59417107 |
| CYP7A1 | rs13251066 | 8 | 59417753 |
| UGT2B17 | rs72551385 | 4 | 69403588 |
| UGT2B17 | rs28374627 | 4 | 69417570 |
| UGT2B15 | rs4148271 | 4 | 69512637 |
| UGT2B15 | rs3100 | 4 | 69512654 |
| UGT2B15 | rs72551389 | 4 | 69512691 |
| UGT2B15 | rs72551390 | 4 | 69512917 |
| UGT2B15 | rs1902023 | 4 | 69536084 |
| UGT2B7 | rs7662029 | 4 | 69961912 |
| UGT2B7 | rs12233719 | 4 | 69962449 |
| UGT2B7 | rs28365062 | 4 | 69964271 |
| UGT2B7 | rs7438284 | 4 | 69964337 |
| UGT2B7 | rs7439366 | 4 | 69964338 |
| UGT2B7 | rs4348159 | 4 | 69972952 |
| UGT2B11 | rs72551394 | 4 | 70066319 |
| UGT2B11 | rs72551395 | 4 | 70070366 |
| UGT2B11 | rs72551396 | 4 | 70070367 |
| UGT2B11 | rs3890590 | 4 | 70078295 |
| UGT2B11 | rs7697037 | 4 | 70079975 |
| UGT2B28 | rs41292949 | 4 | 70160259 |
| UGT2B28 | rs41292951 | 4 | 70160299 |
| UGT2B28 | rs6828191 | 4 | 70160309 |
| UGT2B4 | rs1131878 | 4 | 70345904 |
| UGT2B4 | rs1051752 | 4 | 70346057 |
| UGT2B4 | rs1966151 | 4 | 70346127 |
| UGT2B4 | rs13142440 | 4 | 70346564 |
| UGT2B4 | rs13119049 | 4 | 70346565 |
| UGT2B4 | rs72552707 | 4 | 70351050 |
| UGT2B4 | rs1845555 | 4 | 70355211 |
| UGT2A1 | rs4148311 | 4 | 70454991 |
| UGT2A1 | rs4148310 | 4 | 70455150 |
| UGT2A1 | rs2288741 | 4 | 70455478 |
| UGT2A1 | rs4148304 | 4 | 70460328 |
| UGT2A1 | rs4148301 | 4 | 70462042 |
| UGT2A1 | rs11249454 | 4 | 70499234 |
| UGT2A1 | rs1347046 | 4 | 70513139 |
| CYP2C8 | rs28399518 | 10 | 96796796 |
| CYP2C8 | rs10509681 | 10 | 96798749 |
| CYP2C8 | rs11572103 | 10 | 96818106 |
| CYP2C8 | rs1058930 | 10 | 96818119 |
| CYP2C8 | rs72558196 | 10 | 96826971 |
| CYP2C8 | rs11572080 | 10 | 96827030 |
| CYP2C8 | rs11572066 | 10 | 96829245 |
| UGT8 | rs61733374 | 4 | 115544609 |
| UGT8 | rs4148254 | 4 | 115544713 |
| UGT8 | rs6851610 | 4 | 115544714 |
| UGT8 | rs4148255 | 4 | 115544777 |
| CYP2E1 | rs3813867 | 10 | 135339605 |
| CYP2E1 | rs2031920 | 10 | 135339845 |
| CYP2E1 | rs2070672 | 10 | 135340548 |
| CYP2E1 | rs2070673 | 10 | 135340567 |
| CYP2E1 | rs6413420 | 10 | 135340829 |
| CYP2E1 | rs72559710 | 10 | 135342034 |
| CYP2E1 | rs6413419 | 10 | 135345675 |
| CYP2E1 | rs915909 | 10 | 135347397 |
| CYP2E1 | rs55897648 | 10 | 135351264 |
| CYP2E1 | rs2515641 | 10 | 135351362 |
| CYP2C8 | rs72558195 | 10 | 96824643 |
| UGT1A9 | rs1126803 | 2 | 234526987 |
| UGT1A9 | rs3832043 | 2 | 234580454 |
| UGT1A9 | rs72551329 | 2 | 234580589 |
| UGT1A9 | rs72551330 | 2 | 234580678 |
| UGT1A9 | rs66915469 | 2 | 234581306 |
| UGT1A9 | rs58597806 | 2 | 234581346 |
| UGT1A9 | rs6759892 | 2 | 234601669 |
| UGT1A9 | rs1042707 | 2 | 234601707 |
| UGT1A9 | rs1042708 | 2 | 234601859 |
| UGT1A9 | rs1105880 | 2 | 234601965 |
| UGT1A9 | rs3821242 | 2 | 234637803 |
| UGT1A9 | rs7574296 | 2 | 234638249 |
| UGT1A10 | rs56935833 | 2 | 234545345 |
| UGT1A10 | rs45523834 | 2 | 234545765 |
| UGT1A10 | rs58704432 | 2 | 234545773 |
| UGT1A7 | rs1126802 | 2 | 234545790 |
| UGT1A7 | rs61261057 | 2 | 234590926 |
| UGT1A6 | rs2070959 | 2 | 234602191 |
| UGT1A6 | rs17863783 | 2 | 234602277 |
| UGT1A6 | rs17874942 | 2 | 234621922 |
| UGT1A6 | rs3755320 | 2 | 234622061 |
| UGT1A5 | rs72551334 | 2 | 234621869 |
| UGT1A5 | rs28900406 | 2 | 234681031 |
| UGT1A5 | rs1042709 | 2 | 234681134 |
| UGT1A4 | rs68014726 | 2 | 234656479 |
| UGT1A4 | rs72551335 | 2 | 234622258 |
| UGT1A4 | rs6755571 | 2 | 234627536 |
| UGT1A3 | rs28898617 | 2 | 234637789 |
| UGT1A3 | rs6706232 | 2 | 234637853 |
| UGT1A3 | rs72551338 | 2 | 234638301 |
| UGT1A3 | rs45625338 | 2 | 234637905 |
| UGT1A1 | rs4124874 | 2 | 234665659 |
| UGT1A1 | rs10929302 | 2 | 234665782 |
| UGT1A1 | rs3755319 | 2 | 234667582 |
| UGT1A1 | rs887829 | 2 | 234668570 |
| UGT1A1 | rs4148323 | 2 | 234669144 |
| UGT1A1 | rs72551340 | 2 | 234669155 |
| UGT1A1 | rs72551341 | 2 | 234669457 |
| UGT1A1 | rs72551342 | 2 | 234669462 |
| UGT1A1 | rs72551343 | 2 | 234669558 |
| UGT1A1 | rs72551344 | 2 | 234669631 |
| UGT1A1 | rs72551345 | 2 | 234669759 |
| UGT1A1 | rs62625011 | 2 | 234675738 |
| UGT1A1 | rs72551348 | 2 | 234675807 |
| UGT1A1 | rs72551349 | 2 | 234676519 |
| UGT1A1 | rs72551350 | 2 | 234676567 |
| UGT1A1 | rs72551351 | 2 | 234676568 |
| UGT1A1 | rs55750087 | 2 | 234676880 |
| UGT1A1 | rs72551352 | 2 | 234676883 |
| UGT1A1 | rs72551353 | 2 | 234676905 |
| UGT1A1 | rs72551354 | 2 | 234676924 |
| UGT1A1 | rs72551355 | 2 | 234676982 |
| UGT1A1 | rs72551357 | 2 | 234680912 |
| UGT1A1 | rs72551361 | 2 | 234681090 |
| UGT1A1 | rs10929303 | 2 | 234681416 |
| UGT1A1 | rs1042640 | 2 | 234681544 |
| UGT1A1 | rs8330 | 2 | 234681645 |
| UGT1A1 | rs56059937 | 2 | 234669180 |
| UGT1A1 | rs45627238 | 2 | 234669619 |
| UGT1A1 | rs7586110 | 2 | 234590527 |
| UGT2B15 | rs4148269 | 4 | 69512847 |
